# Alston Virus, a Novel Paramyxovirus Isolated from Bats Causes Upper Respiratory Tract Infection in Experimentally Challenged Ferrets

**DOI:** 10.3390/v10120675

**Published:** 2018-11-28

**Authors:** Rebecca I. Johnson, Mary Tachedjian, Brenton Rowe, Bronwyn A. Clayton, Rachel Layton, Jemma Bergfeld, Lin-Fa Wang, Glenn A. Marsh

**Affiliations:** 1 CSIRO Health and Biosecurity, Australian Animal Health Laboratory, Geelong 3220, Australia; rebecca.johnson@csiro.au (R.I.J.); mary.tachedjian@csiro.au (M.T.); brenton.rowe@csiro.au (B.R.); bronwyn.clayton@ecodev.vic.gov.au (B.A.C.); rachel.layton@csiro.au (R.L.); jemma.bergfeld@unimelb.edu.au (J.B.); 2 Programme in Emerging Infectious Diseases, Duke-NUS Medical School, Singapore 169857, Singapore; linfa.wang@duke-nus.edu.sg

**Keywords:** paramyxovirus, bat-borne, zoonoses

## Abstract

Multiple viruses with zoonotic potential have been isolated from bats globally. Here we describe the isolation and characterization of a novel paramyxovirus, Alston virus (AlsPV), isolated from urine collected from an Australian pteropid bat colony in Alstonville, New South Wales. Characterization of AlsPV by whole-genome sequencing and analyzing antigenic relatedness revealed it is a rubulavirus that is closely related to parainfluenza virus 5 (PIV5). Intranasal exposure of mice to AlsPV resulted in no clinical signs of disease, although viral RNA was detected in the olfactory bulbs of two mice at 21 days post exposure. Oronasal challenge of ferrets resulted in subclinical upper respiratory tract infection, viral shedding in respiratory secretions, and detection of viral antigen in the olfactory bulb of the brain. These results imply that AlsPV may be similar to PIV5 in its ability to infect multiple mammalian host species. This isolation of a novel paramyxovirus with the potential to transmit from bats to other mammalian species reinforces the importance of continued surveillance of bats as a source of emerging viruses.

## 1. Introduction

Bats are the source of multiple zoonotic viruses including severe acute respiratory syndrome (SARS) coronavirus [1], Hendra virus [2], Nipah virus [3], and Marburg virus [4]. In fact, evidence suggests that bats host a greater proportion of zoonotic viruses than any other mammalian order [5], highlighting the importance of identifying novel viruses in bats. Australian pteropid bats are becoming more urbanized and fewer bats are migrating, resulting in a greater chance of contact between bats and humans or domestic animals [6,7]. This increased potential for exposure of non-reservoir hosts to bat-borne viruses leads to the increased probability of infection spillovers occurring [8,9].

Isolation and phenotypic characterization should be critical components of virus discovery programs because the analysis of novel viral sequences is not currently enough to predict the likelihood of that virus causing a zoonotic disease event [10]. The likelihood of viral emergence and sustained human-human transmission is influenced by many factors. In addition to environmental factors and host behaviors, specific viral traits and host-pathogen interactions play important roles. For example, low viral pathogenicity resulting in low host mortality influences opportunities for sustained viral transmission; viral tissue tropism and host immune responses determine shedding at sites relevant to transmission; and the establishment of chronic or latent infection may allow for sustained or recurrent viral shedding [11].

*Paramyxoviridae* is a family of negative strand RNA viruses currently comprising seven genera—*Rubulavirus*, *Henipavirus*, *Respirovirus*, *Morbillivirus*, *Ferlavirus*, *Aquaparamyxovirus* and *Avulavirus* [12]. PCR and virus isolation have been used to identify many paramyxoviruses in bats globally, in particular, henipaviruses and rubulaviruses [13,14]. The genus *Rubulavirus* contains the human pathogens parainfluenza virus 2 (hPIV2) and mumps virus (MuV), as well as bat-borne viruses such as Mapuera and Menangle viruses (MapV and MenPV). Viruses within this genus have a cell attachment glycoprotein with neuraminidase and haemagglutinin capability [15]. In addition to the cell attachment glycoprotein (HN), the rubulavirus genome also encodes a nucleoprotein (N), phosphoprotein (P), V protein, matrix (M) protein, fusion (F) protein and a large polymerase subunit (L) [16]. The unedited P gene transcript encodes the V protein, whereas the addition of two non-templated G residues by co-transcriptional stuttering of the RNA-dependent RNA polymerase is required for the expression of the phosphoprotein [16]. MuV and parainfluenza virus 5 (PIV5) also express a short hydrophobic (SH) protein that has been associated with blockage of the TNFα-mediated apoptosis pathway [17].

PIV5 is most well known as one of the causative agents of Canine Infectious Respiratory Disease Complex (CIRDC), where infection results in self-limiting tracheobronchitis that resolves in 6–14 days when in the absence of any co-infections [18]. Since the discovery of PIV5 in monkey kidney-cell culture in 1954 [19], it has been isolated from a wide range of host species including pigs and cattle [20,21].

Here we describe the isolation of a novel rubulavirus that we have called Alston virus (AlsPV). AlsPV is closely related to PIV5 and was isolated from pteropid bat urine collected in Alstonville, New South Wales in 2011. This is the first isolation of this novel virus. This paper describes the characterisation of this virus in order to confirm its classification as a rubulavirus, as well as to determine its pathogenic potential.

## 2. Materials and Methods

### 2.1. Cell Culture

Cell lines used in the characterization of AlsPV were African Green Monkey (Vero) cells (ATCC), primary *Pteropus alecto* kidney (PaKi) cells [22], Madin–Darby Canine Kidney (MDCK) cells (CSL Ltd., Melbourne, Australia), Madin–Darby Bovine Kidney (MDBK) cells (ATCC), porcine kidney (PK15a) cells (National Animal Disease Centre, Ames, IA, USA) and human cervical (HeLa) cells (ATCC).

With the exception of PaKi cells, all other cell lines were grown in Dulbecco’s Modified Eagle’s Medium (DMEM, Gibco, Grand Island, NY, USA) supplemented with 10% fetal bovine serum (FBS, Gibco), 100 units/mL penicillin, 100 μg/mL streptomycin and 0.25 μg/mL amphotericin B (Antibiotic-Antimycotic, Gibco), and 7.5 mM HEPES (Gibco). PaKi cells were grown in Ham’s F12 Nutrient Mixture (Gibco) supplemented as above for normal cell culture media.

### 2.2. Virus Isolation

For isolations, cells were cultured in Ham’s F12 Nutrient Mixture (Gibco), supplemented as above except for the Antibiotic-Antimycotic that was added at double the normal strength. Virus isolations were conducted using pooled bat urine collected from pteropid bat colonies in Alstonville, New South Wales on the 12 July 2011 and 3 August 2011. Urine collection was conducted as previously described [23]. Urine was clarified, diluted, and incubated with confluent Vero or PaKi cell monolayers as previously described [24]. Cell monolayers were observed for at least one week for evidence of virus-induced cytopathic effect (CPE). Supernatants were further passaged onto fresh Vero and PaKi cell monolayers weekly for another two weeks and observed for signs of CPE.

Isolated paramyxoviruses were initially identified using hemi-nested PCR with degenerate primers following the protocols described previously [25], followed by Sanger sequencing of the PCR products.

### 2.3. Viruses

In addition to Alston virus, viruses used for in vitro analysis included Teviot virus/Bat/2011/Alstonville (TevPV), porcine rubulavirus (PorV), MapV, Tioman virus (TioPV), MenPV and Hendra virus (HeV). The following reagents were obtained through BEI Resources, NIAID, NIH: hPIV2, Greer, NR-3229; and PIV5, 21005-2WR (Tissue Culture Adapted), NR-42515; and MuV, Enders, NR-3846.

The GenBank accession number for the Alston virus sequence is MH972568. Virus sequences used in phylogenetic analysis TevPV (KP271123), TioPV (NP665871), MenPV (AFY09794), PIV5 (YP138518), hPIV2 (X57559), Achimota virus 1 (AchPV1, JX051319), Achimota virus 2 (AchPV2, AFX75118), human parainfluenza virus 4 (hPIV4, AB543336), bat mumps virus (bat-MuV, HQ660095), MapV (EF095490), MuV (NP054714), simian virus 41 (SV41, X64275), PorV (BK005918), Tuhoko virus 1 (ThkPV1, ADI80715), Tuhoko virus 2 (ThkPV2, GU128081), Tuhoko virus 3 (ThkPV3, GU128082), Sosuga virus (SosPV, AHH02041), and HeV (NP047113).

### 2.4. Parainfluenza Virus 5 Sequences

Parainfluenza virus 5 strains used in the analysis of AlsPV included 1168 (KC237064), ZJQ-221 (KX100034), SER (JQ743328), BC14 (KM067467), CC-14 (KP893891), W3A (JQ743318), KNU-11 (KC852177), AGS (KX060176), CPI- (JQ743320), CPI+ (JQ743321), 78524 (JQ743319), H221 (JQ743323), 08-1990 (KC237063), D277 (KC237065), DEN (JQ743322), LN (JQ743324), RQ (JQ743327), MEL (JQ743325), and MIL (JQ743326).

### 2.5. Sequencing

#### 2.5.1. Whole-Genome Sequencing

Supernatant of AlsPV infected Vero cells was prepared for sequencing by ultracentrifugation through a 20% sucrose cushion at 35,000 rpm for 2 h at 4 °C. Total RNA was extracted from the resulting pellet using a Direct-zol RNA Miniprep kit (Zymo, Irvine, CA, USA), including an in column DNaseI digestion, and purified by an RNA Clean and Concentrator kit (Zymo). A REPLI-g WTA Single Cell kit (Qiagen, Venlo, The Netherlands) was utilized for isothermal amplification, followed by processing with a Genomic DNA and Concentrator 10 kit (Zymo). Fragmentation and dual-index library preparation were conducted using Nextera XT DNA Library Preparation kit (Illumina, San Diego, CA, USA), and denatured libraries were sequenced using a 300-cycle MiSeq Reagent kit v2 (Illumina). 100,000 paired-end reads were imported into the VirAMP Galaxy pipeline, trimmed, and assembled using the SPAdes de novo assembly algorithm [26,27]. The genome sequence was iteratively extended by mapping trimmed reads back to the parainfluenza virus 5 genome (NC_006430). Genome ends and regions of high variability were confirmed by Sanger sequencing.

#### 2.5.2. Confirmation of Genome Termini

Ligation of genome ends was used to enable sequencing of the 3′ terminus, adapted from a protocol previously developed for influenza virus sequencing [28]. Genome ends were ligated overnight at 16 °C using 20 U T4 RNA Ligase, 20 U RNasin, 50 μM ATP, 10% PEG8000 and T4 RNA ligase reaction buffer (NEB, Ipswich, MA, USA). Ligation was followed by hemi-nested PCR amplification using a Superscript III One-Step RT-PCR System with Platinum Taq DNA Polymerase (Invitrogen, Carlsbad, CA, USA), then an Expand High Fidelity PCR System (Roche, Basel, Switzerland).

The rapid amplification of cDNA ends (RACE) [29] was required to determine the 5′ terminus, with some adaptations made to the original method. Briefly, viral RNA was reverse transcribed using a virus specific primer and the Superscript III First-Strand Synthesis Supermix (Invitrogen, Carlsbad, CA, USA). Viral cDNA was RNase H digested, followed by processing with a NucleoSpin PCR Clean-up and Gel Extraction kit (Macherey Nagel, Düren, Germany). Viral cDNA was ligated to an oligonucleotide adaptor (5′-GAAGAGAAGGTGGAAATGGCGTTTTGG-3′) overnight at 16 °C using T4 RNA Ligase (NEB) and amplified by hemi-nested PCR using Platinum Taq DNA Polymerase High Fidelity system (Invitrogen) with virus specific primers and an adaptor specific primer. Fragments of the correct size were purified before sequencing by standard Sanger methods.

#### 2.5.3. Amplicon Sequencing

Amplicon sequencing of the RNA editing site within the P gene was conducted on RNA extracted from Vero cells infected with AlsPV for 72 h in triplicate. RNA was reverse transcribed using oligo(dT) primers with Superscript III Reverse Transcriptase (Invitrogen). Fragments containing the RNA editing site were amplified using an Expand High Fidelity PCR System with primers containing Nextera adaptors, (5′-TCGTCGGCAGCGTCAGATGTGTATAAGAGACAGCCCAACCCTCTACTTGGCTTGGATTC-3′) and (5′-GTCTCGTGGGCTCGGAGATGTGTATAAGAGACAGGCCGGGTATCCATCCCTCTCACTG-3′). Controls were included in triplicate to account for the mutation rate of the reverse transcriptase and the polymerase. PCR controls were produced by amplifying pCAGGS constructs containing non-edited fragments with an Expand High Fidelity PCR System. Reverse transcription controls were produced by transfecting constructs containing non-edited fragments into Vero cells for 24 h using Lipofectamine LTX Reagent (Invitrogen), followed by total RNA extraction, reverse transcription and PCR amplification. All PCR products were amplified with Nextera adaptor-specific primers using a HiFi HotStart ReadyMix PCR system (Kapa Biosystems, Wilmington, MA, USA). The DNA library was sequenced using a 600-cycle MiSeq Reagent Kit v3 (Illumina). Data were analyzed using CLC Genomics Workbench 8.5.1 (Qiagen) basic variant detection tool with a minimum frequency output of 0.05%. The prevalence of editing was standardized per 100,000 reads.

### 2.6. Virus Quantification

10-fold serial dilutions of virus stocks were combined with Vero cells in 96-well plates to determine the 50% tissue culture infectious dose per milliliter (TCID_50_/mL). Virus titers were calculated using the Reed–Muench method [30].

### 2.7. Growth Kinetics Assay

Comparative growth analysis in multiple mammalian cell lines was conducted as described previously [14]. Briefly, confluent cells were inoculated with AlsPV at an MOI of 0.01 and incubated for 1 h at 37 °C. Cells were washed four times with PBS and cell culture media was added. Infected cells were incubated at 37 °C and aliquots were taken every 24 h for 6 days. Virus titers were determined as above. Virus titers were compared by two-way ANOVA followed by Bonferroni adjustment using GraphPad Prism 5 (LaJolla, CA, USA).

### 2.8. Immunofluorescence Assay

Confluent Vero cells were infected with virus at an MOI of 0.01 and incubated for 2–3 days at 37 °C. Cells infected with Hendra virus at an MOI of 0.5 were incubated at 37 °C for 24 h under BSL4 conditions. Infected cells were fixed with ice-cold methanol for 15 min, or 30 min for cells infected with Hendra virus, before blocking with 1% BSA at 37 °C for 30 min. Cells were incubated with primary antibody for 1 h at 37 °C and washed four times with PBS-T. Following this, cells were incubated for 1 h at 37 °C with a secondary antibody, either Protein A-Alexa Fluor 488 or anti-rabbit-Alexa Fluor 488, and DAPI. Cells were washed four times with PBS-T before imaging using an EVOS FL Cell Imaging System (Life Technologies, Carlsbad, CA, USA).

### 2.9. Neutralisation Assay

Paramyxovirus sera were first inactivated by incubating at 56 °C for 35 min. Cell culture media containing 100 TCID_50_ of AlsPV or other paramyxoviruses were incubated with two fold dilutions of various paramyxovirus sera or AlsPV ferret sera for 30 min at 37 °C. A suspension of 2 × 10^4^ Vero cells was added to each well, and plates were incubated for 5–7 days and then assessed for the presence of CPE. Neutralizing titers were calculated using the Reed–Muench method as described previously as the reciprocal of the highest dilution of serum at which the infectivity of 100 TCID_50_ of virus is neutralized in 50% of the wells [31].

### 2.10. Australian Flying Fox Serology

Australian pteropid bat sera, collected between 1999 and 2012, were inactivated by treating at 56 °C for 35 min. Sera at a 1:10 dilution were incubated in quadruplicate with 100 TCID_50_ AlsPV for 45 min before the addition of a suspension of 2 × 10^4^ Vero cells per well. Plates were incubated for 7 days before being assessed for the presence of virus-induced CPE.

### 2.11. Animal Experiments

All procedures were approved by the CSIRO Australian Animal Health Laboratory Animal Ethics Committee. Study 1, project number 1814, was approved in August 2016. Study 2, project number 1865, was approved in June 2017.

#### 2.11.1. Study 1

Female ferrets (*n* = 3) were exposed oronasally to 7 × 10^5^ TCID_50_ AlsV in 1 mL sterile PBS. Adult female BALB/c mice aged between 6–9 months (*n* = 5) and juvenile (8 week old) female BALB/c mice (*n* = 5) were exposed intranasally to 2 × 10^4^ TCID_50_ AlsV in 30 μL sterile PBS whilst under anesthesia (ferrets—0.05 mg/kg medetomidine and 5 mg/kg ketamine; mice 1 mg/kg medetomidine and 75 mg/kg ketamine). Ferrets sourced from the CSIRO Werribee Animal Facility were approximately one year old and had a mean weight of 890 g. Mice were provided by the CSIRO Australian Animal Health Laboratory Small Animal Facility. Virus stock used for animal challenge was diluted 1/10 to reduce the risk of adverse reactions such as laryngospasm during virus challenge, while still maintaining a high enough dose to maximize the likelihood of infection. The challenge doses of inocula were confirmed by back titration.

Animals were monitored for clinical signs of disease for 21 days following challenge. Oral swabs, nasal washes, rectal swabs and EDTA-treated whole blood samples were collected from ferrets on days 3, 5, 7, 10, and 14 days post-infection and again at euthanasia on day 21. Sera were also collected from ferrets on day 7 onward and urine was collected at euthanasia. Weight, rectal temperature, and body temperature measurements were collected from ferrets at each sampling event. Weight and microchip temperature were measured daily for mice.

Tissues collected at euthanasia for assessment by both virus isolation and qRT-PCR were lung, kidney, spleen, brain (olfactory bulb plus 2 mm caudal) and liver from mice; and lung, kidney, spleen, brain (olfactory bulb plus 2 mm caudal), liver and retropharyngeal lymph node from ferrets. Tissues were fixed in a 10% neutral buffered formalin for histology analysis.

#### 2.11.2. Study 2

Female ferrets (*n* = 12), approximately one year old with a mean weight of 800 g, were exposed to 7 × 10^5^ TCID_50_ AlsV as for study 1. Three ferrets were euthanized on each of days 3, 5, 7, and 10 post-inoculation, based on random allocation of a time point for euthanasia.

Microchip temperature was recorded daily from all animals following challenge. On the day of euthanasia, oral swabs, nasal washes, rectal swabs, urine, and blood were collected from each ferret, and weight and rectal temperature measurements recorded. Tissues collected for the detection of virus by isolation and qRT-PCR were brain (olfactory bulb plus 2 mm caudal), nasal turbinates, tonsil, trachea, peripheral lung, hilar lung, spleen, kidney, liver, heart, small intestine, large intestine, bronchial lymph node and retropharyngeal lymph node. Tissues were also stored in 10% neutral buffered formalin for histology analysis.

#### 2.11.3. Analysis of Animal Infection Study Samples

RNA was extracted from swab, EDTA-blood and homogenized tissue samples using MagMAX-96 Viral RNA Isolation Kit (Applied Biosystems, Foster City, CA, USA) and analyzed by quantitative RT-PCR. RNA was amplified with AgPath-ID One-Step RT-PCR Reagents (Applied Biosystems) using primers and probe targeting a region in the viral nucleocapsid gene—AlsPV-N287F (5′-AATCCCGAGCTACGTTCAAAACT-3′), AlsPV-N360R (5′-TGGGAGTCACGAGCTCCATT-3′), AlsPV N-311-FAM (5′-FAM-CTGCTATTTTGCCTACGCATTGTGCTGA-TAMRA-3′)—and 18S as an internal control—18S-F (5′-GGCCCTGTAATTGGAATGAGTCCA-3′), 18S-R (5′-GCTGGAATTACCGCGGCT-3′), 18S-VIC (5′-VIC-TGCTGGCACCAGACTTGCCCTC-TAMRA-3′). Reactions were incubated at 45 °C for 10 min and 95 °C for 10 min, and cycled 40 times at 95 °C for 15 s and 60 °C for 45 s on a QuantStudio6 (Applied Biosystems). Copy numbers were calculated using standard curves generated by serially diluting RNA transcribed from control DNA plasmids. To facilitate data interpretation, a copy number of 5 in both qRT-PCR replicates, correlating with a *C*_T_ value of 40 (study 1) or 37.4 (study 2), was used as the minimum of detection. Viral N gene copy numbers in tissue samples were standardized to 18S expression (per 10^10^ copies of 18S RNA). Viral N gene copy numbers in shedding samples were calculated per milliliter of sample. Results were analyzed using QuantStudio6 software. For virus isolation and titration, 10-fold serial dilutions of samples were made in 96-well plates. A suspension of 2 × 10^4^ Vero cells was added to each well and plates were incubated at 37 °C for 7 days before assessing for signs of CPE. Titers were calculated using the Reed–Muench formula [30].

#### 2.11.4. Histology

Tissues were fixed in 10% neutral buffered formalin, and then trimmed and processed using routine histological methods as previously described [32]. Sections were assessed for the presence of histopathological lesions and viral antigen following routine hematoxylin and eosin staining and immunohistochemical staining using rabbit antibodies raised against a recombinant AlsPV N protein peptide (Genscript, Piscataway, NJ, USA).

### 2.12. Antibodies

AlsPV polyclonal antibodies were generated in rabbits by Genscript (USA) using the peptide RQQGRINPRYLLQP from the AlsPV N protein. AlsPV ferret antisera produced in the animal infection trials described in this study were also utilized. Other primary antibodies included rabbit or pig antisera against MenPV (AAHL), rabbit or pig antisera against TioPV (AAHL), TevPV ferret antisera (AAHL), rabbit or horse antisera against HeV (AAHL), PorV rabbit antisera (AAHL) and MapV rabbit antisera (AAHL). Polyclonal anti-PIV5, 21005-2WR (antiserum, guinea pig), NR-3232, polyclonal anti-mumps virus, Enders (antiserum, guinea pig), NR-4019 and polyclonal anti-hPIV2, Greer, (antiserum, guinea pig), NR-3231 were obtained through the NIH Biodefense and Emerging Infections Research Resources Repository, NIAID, NIH.

### 2.13. Protein Prediction

Membrane topology of AlsPV proteins was predicted using Phobius as described in [33].

### 2.14. Sialidase Assay

Confluent Vero cell monolayers were treated with 15 mU of *Arthrobacter ureafaciens* neuraminidase for 2 h at 37 °C in cell culture media. Untreated and neuraminidase-treated cells were washed twice with PBS and incubated with AlsPV or PIV5 (MOI 2) in duplicate for 1 h. The cells were then washed four times with PBS and incubated for 24 h in cell culture media, before fixing and immunofluorescence staining as above. Fluorescent cells were counted in nine fields of view per well using an ImageXpress Micro XLS Widefield High-Content Analysis System (Molecular Devices, San Jose, CA, USA) and compared to untreated infected cells. The relative number of infected cells was compared by one-way ANOVA followed by Dunnett’s multiple comparison test (compared to untreated cells) using GraphPad Prism 5.

## 3. Results

### 3.1. Isolation of a Novel Bat-Borne Rubulavirus

Bat urine samples were collected from Alstonville, New South Wales in July and August of 2011. Inoculation of the bat urine onto primary bat kidney (PaKi) cells and Vero cells resulted in the isolation of a novel paramyxovirus. Cytopathic effect was initially observed in PaKi cells 18 days post inoculation. RT-PCR and Sanger sequencing resulted in a 500 nt fragment of L gene that was identical to a L gene fragment that had been previously detected by PCR in urine collected from grey-headed flying foxes, *Pteropus poliocephalus*, in Geelong, Victoria in 2010 (KM359175.1). In that instance, however, the virus was unable to be cultured [34]. Whole genome sequencing of PaKi cell supernatant confirmed the presence of a novel paramyxovirus and the name Alston virus (AlsPV) was chosen based on the location of the source bat colony. No other virus or bacteria were detected in the supernatant by next generation sequencing.

### 3.2. Analysis of the AlsPV Whole-Genome Sequence

**Figure 1:**
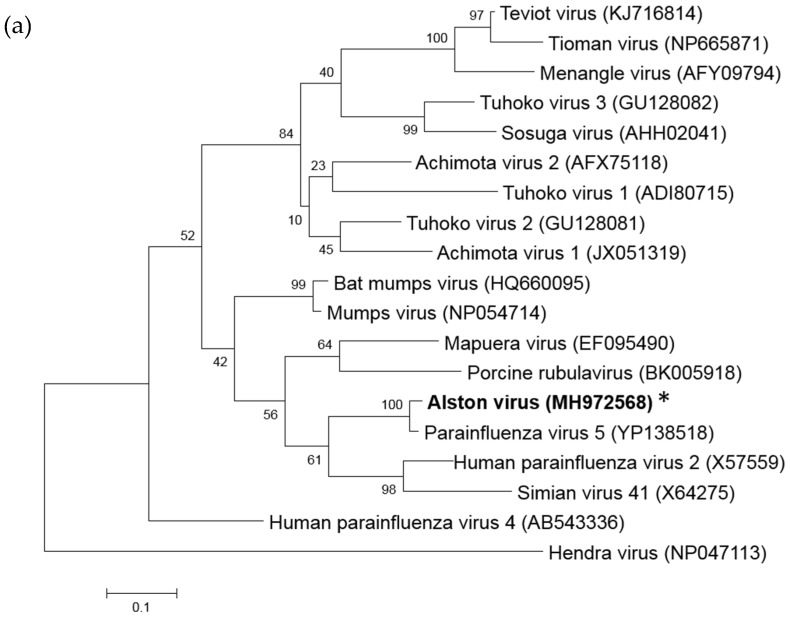

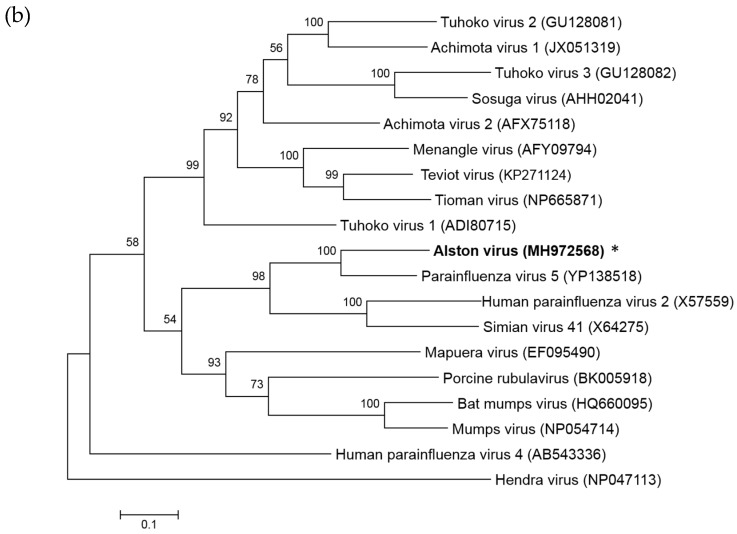

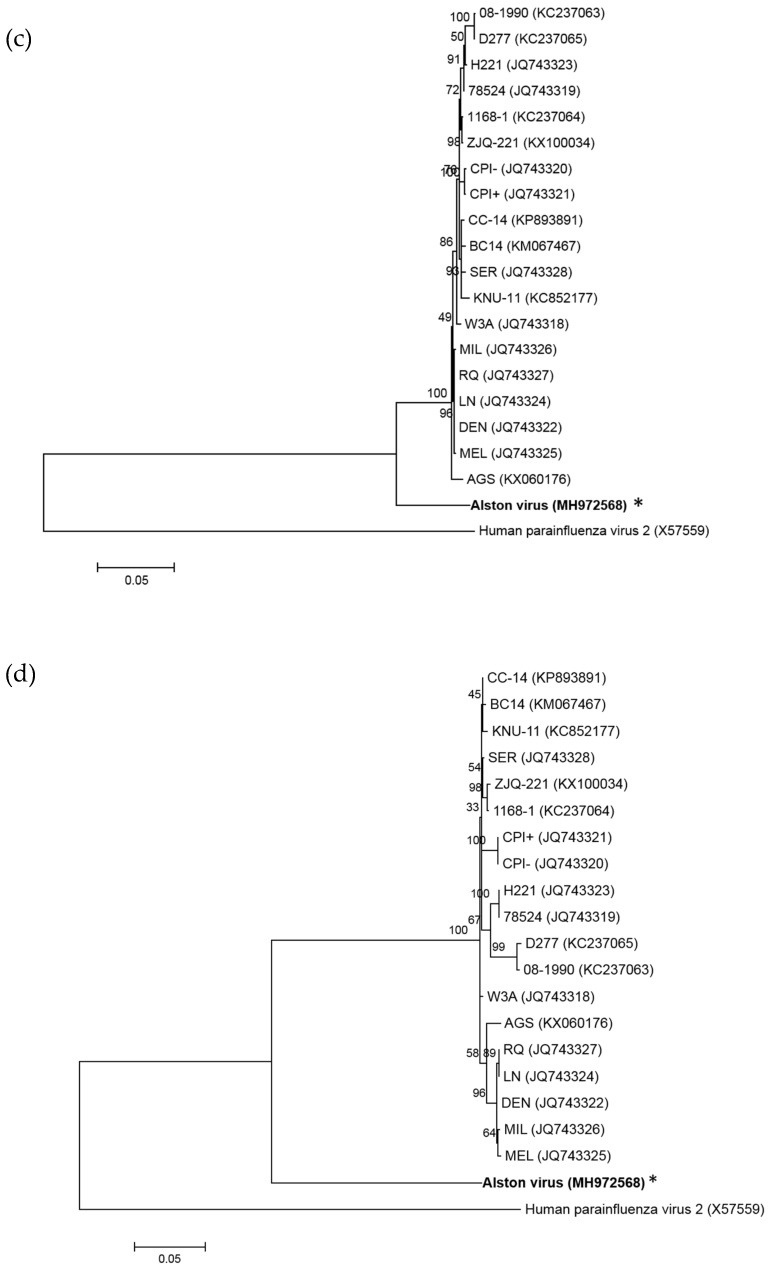
Phylogenetic analysis of rubulaviruses. (**a**) L protein or (**b**) N gene of rubulaviruses with Hendra virus as an outgroup. (**c**) L protein or (**d**) N gene of multiple parainfluenza virus 5 strains, AlsPV and human parainfluenza virus 2. All trees are maximum-likelihood tree reconstructed with MEGA 6.06, bootstrapping to 1000 replicates. AlsPV is highlighted in bold and with an asterisk.

The whole genome sequence of AlsPV was assessed for the presence of paramyxovirus motifs and features, as well as phylogenetically analyzed to determine its classification as a novel virus. Whole genome sequencing revealed that AlsPV is a novel virus from the genus *Rubulavirus* (Figure 1). The genome of AlsPV is 15270 nucleotides long with a GC content of 41.6%. The coding percentage is 92.2%, which is the average coding percentage of the paramyxoviruses, not including members of the genus *Henipavirus* [35]. It has a 55 nt leader sequence and a 31 nt trailer sequence that have been confirmed using a combination of 5′ rapid amplification of cDNA ends (RACE) and sequencing across ligated genome ends.

Phylogenetic analysis indicated that AlsPV is most closely related to PIV5 (Figure 1). The lengths of the genomes and genes are highly conserved between PIV5 and AlsPV (Table 1), as well as the sequences of the gene boundaries and the length of untranslated regions and intergenic regions (Table S1). Furthermore, comparison of coding regions of PIV5 and AlsPV revealed nucleotide identities between 63–81% and amino acid (aa) identities between 61–93% (Table 2). Across the whole genome, including non-coding regions, the nucleotide identity was found to be 74%.

### 3.3. Analysis of Deduced Amino Acid Sequences

#### 3.3.1. SH

The short hydrophobic (SH) gene is only encoded by the genomes of MuV and PIV5, so the AlsPV genome was assessed for the presence of an additional open reading frame in between the F gene and the HN gene. AlsPV was found to have this additional open reading frame with the capacity to express an SH protein of 44 aa in length. Despite lower similarity with PIV5 in this gene compared to the rest of the coding regions, the SH protein of AlsPV is predicted to have the same cell surface orientation as the SH protein of PIV5 [36]. The AlsPV SH protein is predicted to be a class II integral membrane protein with 16–19 cytoplasmic N-terminal residues and 1–5 C-terminal residues. It is therefore possible that the SH protein of AlsPV has the same anti-apoptotic functions as the SH proteins of PIV5 and MuV.

#### 3.3.2. HN

**Figure 2:**
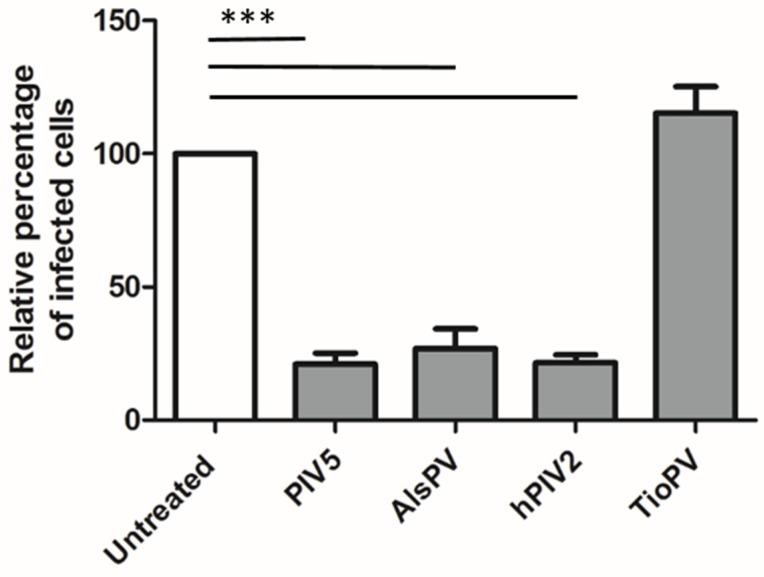
Effect of *Arthrobacter ureafaciens* neuraminidase treatment on AlsPV, PIV5, hPIV2 and TioPV infection of Vero cells. Infected cells were counted and averaged across nine fields of view and compared to infected but untreated cells. TioPV was included as a control because it lacks the NRKSCS motif and was therefore not expected to bind sialic acid. Values represent a percentage of the number of infected cells counted in untreated samples. Error bars represent standard error of the mean. Significance calculated by one-way ANOVA followed by Dunnett’s multiple comparison test (compared to untreated cells). *** represents a *p* value of <0.001. *n* = 2 independent experiments.

Recently, a number of bat-borne rubula-like viruses have been isolated with attachment glycoproteins that appear to lack hemagglutinin and neuraminidase activity, however, the AlsPV HN gene contains important motifs and residues associated with neuraminidase function, in particular, the NRKSCS motif [37,38]. Treatment of Vero cells with a broad acting sialidase prior to infection resulted in significant reduction in the ability of the virus to infect cells (Figure 2). It is likely that, similar to PIV5 and multiple other rubulaviruses, sialic acid is the main cellular receptor used for attachment to host cells.

#### 3.3.3. RNA Editing of the P Gene

Similar to other rubulaviruses, the unedited transcript of the AlsPV P gene encodes the V protein and insertion of two guanine residues at the editing site results in the expression of the P protein. Amplicon sequencing was used to determine the prevalence of edited transcripts per 100,000 reads. On average, 79.63% of the transcripts were unedited or had the addition of three G residues resulting in expression of the V protein. Transcripts encoding the P protein through the addition of two or five G residues occurred 19.09% of the time. In 1.28% of transcripts, the addition of one or four G residues encoded a putative W protein. This totaled an average editing frequency of 21.8%. Insertions of up to eight G residues could be detected at the editing site, although at low frequency.

### 3.4. AlsPV is Antigenically Related to PIV5

**Figure 3:**
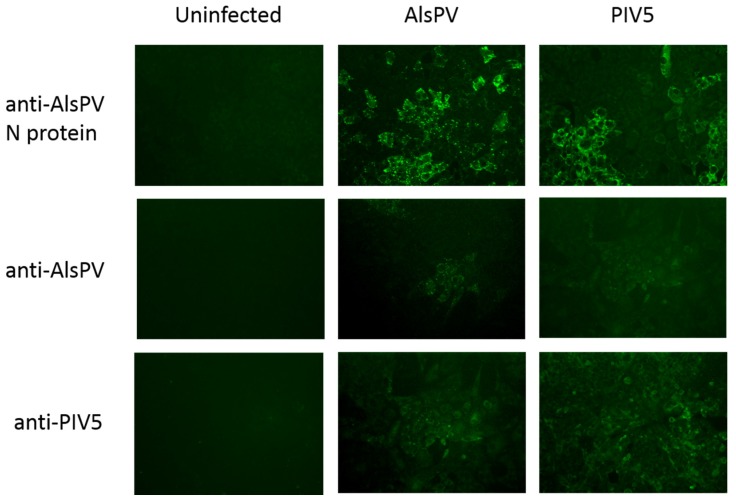
Antigenic cross-reactivity between PIV5 and AlsPV by immunofluorescence assay. Vero cells were infected AlsPV or PIV5 and stained with either rabbit sera raised against an N protein peptide of AlsPV, ferret sera resulting from infection with AlsPV, or anti-PIV5 guinea pig sera (magnification ×20).

In order to add evidence to its classification as a novel rubulavirus, the antigenic relatedness of AlsPV to other paramyxoviruses was determined by immunofluorescence assay (IFA). Analysis of antigenic relatedness by immunofluorescence assay showed cross-reactivity between AlsPV and PIV5 (Figure 3). There were low levels of cross-reactivity with hPIV2, MuV, MenPV, TioPV and TevPV when using antisera against the AlsPV N protein. No cross-reactivity was detected for HeV, PorV or MapV.

AlsPV was neutralized by low dilutions of PIV5 antisera, however, AlsPV antisera were unable to neutralise PIV5 (Table 3). Additional higher titer PIV5 sera are needed to further investigate whether this is truly only a one-way neutralization. There was no cross-neutralization between AlsPV and any other tested virus; MenPV, TioPV, TevPV MuV, hPIV2, MapV, PorV and HeV.

### 3.5. Growth Analysis of AlsPV in Mammalian Cell Lines

**Figure 4:**
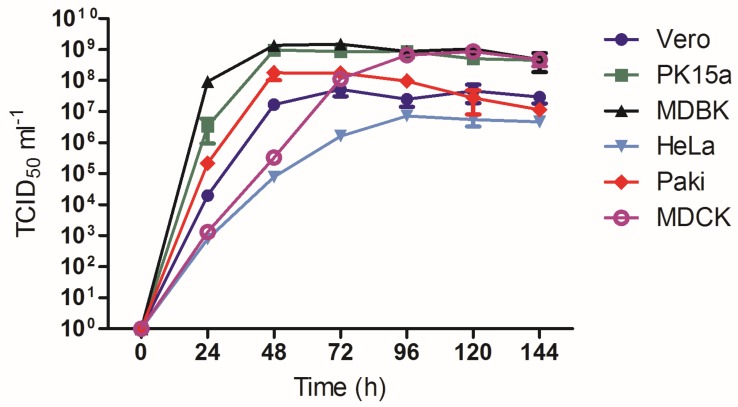
Growth kinetics of AlsPV in multiple mammalian cell lines. Mammalian cell lines were infected with AlsPV at MOI 0.01 for 1 h in triplicate. Cells were washed and aliquots were collected every 24 h for 6 days. The TCID_50_/mL was determined by virus titration. Error bars represent standard error of the mean.

Comparative growth analysis of AlsPV in multiple mammalian cell lines was conducted as a preliminary indication of the potential for AlsPV to infect other mammalian species. AlsPV was found to grow to high titers in all tested mammalian cell lines, with AlsPV growth plateauing at significantly higher titers in MDBK, MDCK and PK15a cells than in HeLa, PaKi and Vero cells when assessed by two-way ANOVA followed by Bonferroni adjustment. (Figure 4, Table S2). These higher titers may be a consequence of the virus not causing significant damage to the cells as only minimal cytopathic effect (CPE) was observed in MDCK, MDBK, and PK15a cells.

### 3.6. AlsPV Neutralising Antisera Are Prevalent in Grey Headed Flying Foxes

To determine the potential exposure rate of Australian flying foxes to AlsPV, 120 pteropid bat sera samples were tested by neutralization assay. Only ~8% (10/120) of total tested pteropid bat sera samples neutralized the growth of AlsPV, however, 25% (5/20) of *P. poliocephalus* sera neutralized AlsPV, suggesting it may be the primary host species for AlsPV (Table 4).

### 3.7. Animal Infection Studies

#### 3.7.1. AlsPV Is Shed in Ferret Respiratory Secretions

Animal infection studies in ferrets and mice were completed to attempt to determine the pathogenic potential of AlsPV in mammalian species. Mice remained clinically normal, with no evidence of fever, weight loss or behavioral changes, until scheduled euthanasia at 21 days post infection, and there was no evidence of seroconversion. Low copy numbers of viral RNA could be detected by qRT-PCR in the brains of two mice, one adult (169 copies of AlsPV N per 10^10^ copies of 18S RNA) and one juvenile (5 copies of AlsPV N per 10^10^ copies of 18S RNA), but there was no evidence of viral antigen or inflammation when the brain was assessed by histopathology.

**Figure 5:**
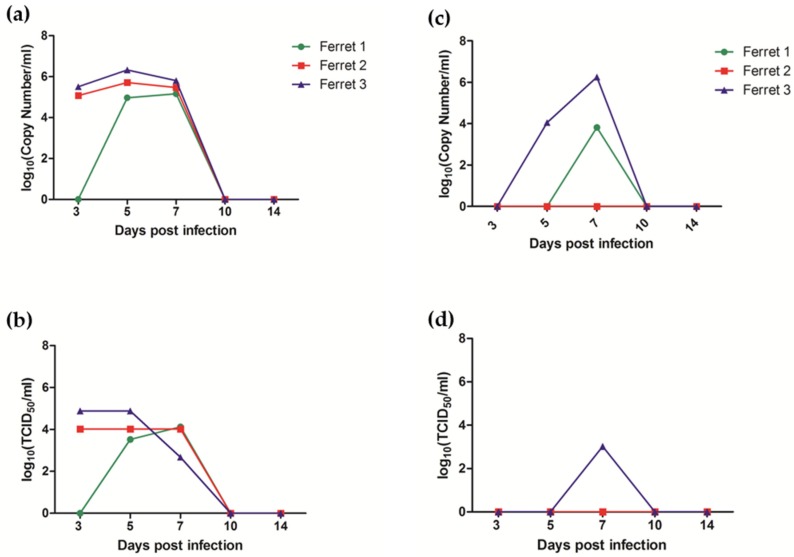
Shedding of AlsPV in ferret respiratory secretions following oronasal exposure. Graphs present log transformations of the (**a**) copy number of AlsPV N gene per ml of nasal wash sample, (**b**) titer of AlsPV isolated from nasal wash, (**c**) copy number of AlsPV N gene per ml of oral swab sample, and the (**d**) titer of AlsPV isolated from oral swabs.

Ferrets also remained clinically normal with no fever, weight loss or behavioral changes following exposure to AlsPV, however, infectious virus was isolated from oral swab and nasal wash samples collected during acute infection (Figure 5). At 10 days post infection, oral and nasal shedding of virus could no longer be detected, correlating with the first detection of seroconversion in one of the three ferrets. Virus neutralization assays showed that all three ferrets had seroconverted by day 14 (Table 5). The shedding samples indicated that virus was replicating, potentially in the upper respiratory tract, but by euthanasia on day 21 no viral RNA could be detected in any tissues by qRT-PCR. Viral RNA could not be detected in ferret urine or blood.

#### 3.7.2. AlsPV Infects the Ferret Upper Respiratory Tract and Olfactory Lobe of the Brain

**Figure 6:**
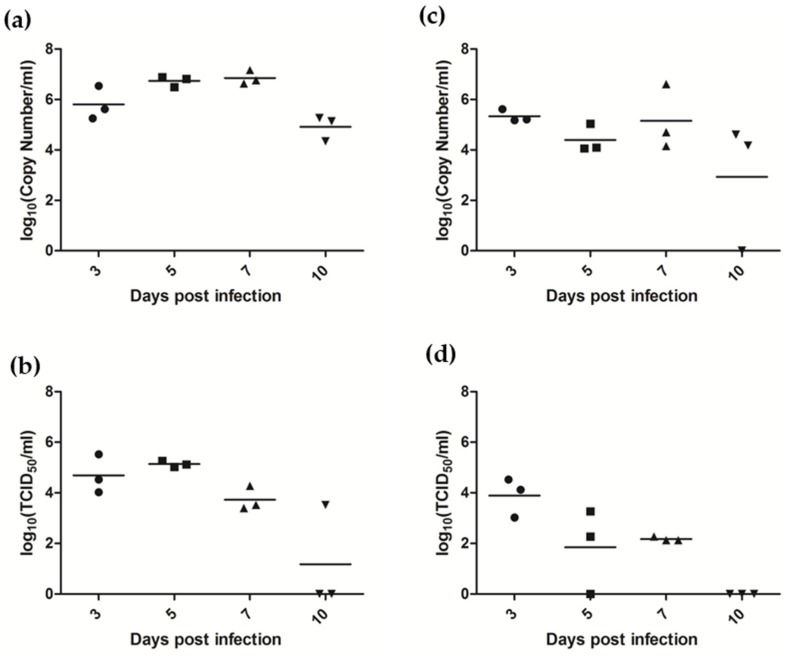
Shedding of AlsPV in ferret respiratory secretions following oronasal exposure. Ferrets were sampled prior to euthanasia, either 3, 5, 7 or 10 days post infection. Graphs present log transformations of the (**a**) copy number of AlsPV N gene per ml of nasal wash sample, (**b**) titer of AlsPV isolated from nasal wash, (**c**) copy number of AlsPV N gene per ml of oral swab sample, and the (**d**) titer of AlsPV isolated from oral swabs.

A second animal infection study was conducted to determine sites of virus replication during the acute stages of infection. As observed in the previous experiment, ferrets remained clinically normal and no viral RNA could be detected in ferret urine or blood. Seroconversion was detected in one of three ferrets euthanized on day 10. Oral and nasal virus shedding was similar to what was observed in study 1 but virus was shed at higher titers and for a prolonged time (Figure 6). Viral RNA was detected in the rectal swab of one ferret euthanized 5 days post infection (3.4 × 10^4^ copies of AlsPV N per mL). This ferret also had low copy numbers of viral RNA detected in the small intestine (41 copies of AlsPV N per 10^10^ copies of 18S RNA).

**Figure 7:**
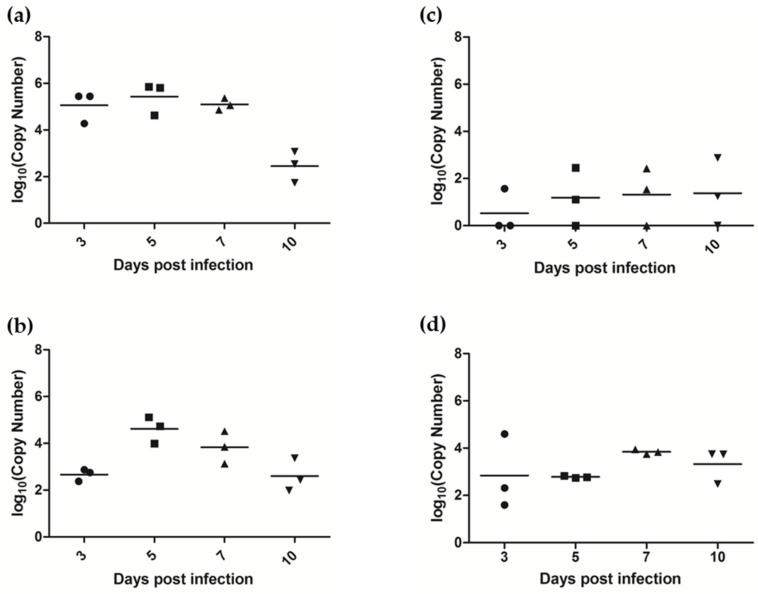
Detection of viral RNA in ferret tissues at euthanasia. Graphs represent log transformations of the copy number of AlsPV N gene RNA per 10^10^ copies of 18S rRNA detected in (**a**) nasal turbinates (**b**) tonsils (**c**) retropharyngeal lymph nodes (**d**) olfactory bulb of the brain.

Viral RNA was detected in a number of tissues at euthanasia (Table 6). In particular, viral RNA was detected in the olfactory bulb of the brain, nasal turbinates and palatine tonsils of all twelve ferrets (Figure 7). Virus was reisolated from 9/12 nasal turbinate samples, 4/12 tonsil samples and from 2/12 olfactory bulb samples, suggesting that live virus was present in these tissues. Low titers of viral RNA could sometimes be detected in the retropharyngeal lymph node, the main draining lymph node of the upper respiratory tract, as well as the trachea and lung. Occasionally, viral RNA could be detected in other organs at low titers.

Although many of the PCR positive samples, such as the nasal turbinates, tonsils, and most olfactory lobes, were unavailable for histology, the olfactory lobe of the brains collected from ferrets euthanized on day 10 were assessed by routine histology. Two out of three olfactory lobes had evidence of inflammation consistent with viral infection, in addition to the presence of low amounts of viral antigen (Figure S1). No other organs, including the remainder of the brain, showed signs of inflammation or viral antigen.

## 4. Discussion

Analysis of pteropid bat urine collected in northern New South Wales in 2011 has led to the isolation of a novel rubulavirus that we have named Alston virus (AlsPV). Phylogenetic and antigenic analyses indicated that this virus is closely related to PIV5. However, in comparison to the observed variation between AlsPV and PIV5, 26% across the whole genome, isolates of PIV5 are almost identical despite being isolated from a range of host species, geographical locations and over multiple decades [39]. In fact, variability of only 7.8% is observed between strains of PIV5, with an average pairwise difference of only 2.1% at the nucleotide level [39]. Given the level of genetic change seen with AlsPV compared to PIV5 (greater than 20% diversity), we propose that AlsPV is a new species of rubulavirus and not a new strain of PIV5.

Despite proposing that AlsPV is a novel species, AlsPV and PIV5 share phenotypic similarities. Experimental intranasal infection of ferrets with PIV5, similar to the infection studies described here, demonstrated variable results ranging from no clinical symptoms to mild cough with minimal lesions in the nasal cavities and upper trachea [40,41]. Antigen could only be detected in the trachea with no evidence of virus in the lungs or the brain [40,41], although it is not known if the olfactory bulb was specifically investigated. Neurological symptoms have been observed in ferrets experimentally infected with PIV5, but only after intracerebral injection as the route of infection. In addition to these in vivo similarities, PIV5 and AlsPV are similar in that they utilize sialic acid as a receptor for cell entry (49), grow to high titers in multiple mammalian cell lines with minimal cytopathic effect (229, 230) and encode an SH gene (49). The multiple similarities between the two viruses indicate that, despite the lack of clinical disease in ferrets and mice, AlsPV may also have the potential to infect other mammalian species.

The findings presented here suggest that AlsPV causes an upper respiratory tract infection in ferrets that is followed by infection of the olfactory pole of the brain. It is likely that from the nasal turbinates, the virus has access to the olfactory neurons that extend from the olfactory bulb through the cribriform plate and into the olfactory neuroepithelium in the nasal cavity [42], but further histopathological examination would be required to confirm this hypothesis. This allows direct access for the virus to disseminate through the central nervous system. However, AlsPV was not detected in brain tissue beyond the olfactory bulb. It is likely that the innate immune response had a role in preventing the spread of AlsPV throughout the CNS [42,43]. Further investigation into the persistence of AlsPV is required, particularly as reduced cytopathic effect was observed during infection of some mammalian cell lines with AlsPV and viral RNA was detected in the olfactory pole of mouse brains at 21 days post infection.

The timing of detection of virus shedding in respiratory secretions of ferrets suggests that virus replication peaked around 5–7 days post inoculation. Although there was some variability between shedding in the first and second animal infection studies, it may have been due to variation between cohorts of outbred ferrets. The presence of virus in oronasal shedding samples and in the upper respiratory tract suggests that AlsPV replicates in tissues that are relevant to virus transmission, although transmission studies are required to confirm if transmission occurs in ferrets. Further experiments are also required to determine the effect of the route of virus challenge and if oronasal infection with lower doses of AlsPV still results in subsequent infection of the olfactory nerve.

Although AlsPV neutralizing antibodies were found at a low overall prevalence in Australian pteropid bat sera, analysis of individual pteropid species indicated that the *Pteropus poliocephalus* flying foxes may be the primary reservoir host. This species was observed in the colony in Alstonville as well as in Geelong where AlsPV was detected by PCR. The high proportion of positive sera samples from grey headed flying foxes, combined with the increasing urban habituation of pteropid bats [6], suggest that there is risk of exposure and potential transmission of AlsPV to non-pteropid mammalian species.

## Figures and Tables

**Table 1:** Comparison of AlsPV and PIV5 coding sequence and protein lengths. Differences between AlsPV and PIV5 are in bold.

Gene/Feature	Coding Region	Virus	Length (nt)	Length (aa)
**3′ leader**		AlsPV	55	
		PIV5	55	
**N**		AlsPV	1530	509
		PIV5	1530	509
**P**	V	AlsPV	669	222
		PIV5	669	222
	P	AlsPV	1179	392
		PIV5	1179	392
	W	AlsPV	516	171
		PIV5	516	171
**M**		AlsPV	1134	377
		PIV5	1134	377
**F**		AlsPV	1629	542
		PIV5	**1590**	529 *
**SH**		AlsPV	135	44
		PIV5	135	44
**HN**		AlsPV	1698	565
		PIV5	1698	565
**L**		AlsPV	6768	2255
		PIV5	6768	2255
**5′ Trailer**		AlsPV	31	
		PIV5	31	

* The F protein length of PIV5 varies from 529 aa to 551 aa.

**Table 2:** Nucleotide and amino acid sequence identities between AlsPV and PIV5. Identities calculated by ClustalW alignment in Geneious 10.1.3.

	N	P	V	W	M	F	SH	HN	L
**Nucleotide**	76	79.4	80.6	76.9	76.6	72.5	63	71	76.7
**Amino acid**	91	86	89	86.6	93	85	61	81	92

**Table 3:** Neutralization titers of ferret AlsPV antisera, guinea pig PIV5 antisera and guinea pig hPIV2 antisera against AlsPV, PIV5 or hPIV2 infection. Neutralization titers were calculated using the Reed–Muench method, described as the reciprocal of the highest dilution of serum at which the infectivity of 100 TCID_50_ of virus is neutralized in 50% of the wells. Results from matched virus-serum pairs are in bold.

	Sera from	AlsPV	PIV5	hPIV2
Infected with				
AlsPV		**202**	14	<10
PIV5		<10	**160**	<10
hPIV2		<10	<10	**226**

**Table 4:** Prevalence of neutralizing antibodies to AlsPV in Australian flying foxes. Sera from pteropid bats collected in Queensland between 1999 and 2007, or Victoria in 2012 were diluted 1/10 and incubated with 100 TCID_50_ AlsPV for 45 min before the addition of Vero cells.

	No. Positive	Percentage Positive
Pteropus sp.*	4/59	6.8
*P. scapulatus*	0/15	0
*P. alecto*	1/26	3.8
*P. poliocephalus*	5/20	25
Total	10/120	8.3

* Pteropid bat sera collected in Queensland between 1999 and 2007, species not recorded.

**Table 5:** Neutralizing antibody titers from AlsPV-infected ferrets. Neutralizing titers were calculated using the Reed–Muench method as described previously [31].

	Day 7	Day 10	Day 14	Day 21
**Ferret 1**	<10	<10	13	101
**Ferret 2**	<10	13	13	40
**Ferret 3**	<10	<10	32	202

**Table 6:** Detection of AlsPV N gene RNA and isolation of AlsPV from tissues collected from AlsPV-infected ferrets.

		Dpi 3			Dpi 5			Dpi 7			Dpi 10		
	Ferret #	1	2	3	4	5	6	7	8	9	10	11	12
	
Nasal turbinates		+/+	+/+	+/+	+/+	+/+	+/+	+/+	+/+	+/+	+/−	+/−	+/+
Tonsil		+/+	+/−	+/−	+/+	+/+	+/+	+/−	+/−	+/−	+/−	+/−	+/−
Retropharyngeal L.N.		+/−	-	-	+/−	-	+/−	+/−	-	+/−	+/−	-	+/−
Trachea		-	-	-	-	-	+/−	+/−	-	-	-	-	+/−
Lung (hilus)		-	-	-	+/−	-	+/−	-	-	-	-	-	-
Lung (peripheral)		-	-	-	+/−	-	-	-	-	+/−	-	-	-
Bronchial L.N.		-	-	+/−	-	-	-	-	-	-	+/−	+/−	-
Heart		-	-	-	-	-	+/−	-	-	-	-	-	-
Liver		-	-	-	-	-	-	-	-	-	-	-	-
Kidney		-	-	-	-	-	-	-	-	-	-	-	-
Spleen		-	-	-	-	-	-	-	-	-	-	-	-
Brain		+/+	+/−	+/−	+/−	+/−	+/−	+/−	+/−	+/+	+/−	+/−	+/−
Small intestine		-	-	-	-	-	+/−	-	-	-	-	-	-
Large intestine		-	-	-	-	-	-	-	-	-	-	-	-

Virus detection in tissue samples at euthanasia, RNA/virus isolation. +/+ indicates the sample was positive by both qRT-PCR and virus isolation; +/− indicates the sample was only positive by qRT-PCR and not by virus isolation; - indicates virus was not detected by qRT-PCR therefore virus isolation was not attempted. Dpi, days post infection; L.N., lymph node; #, number.

## Supplementary Materials

Supplementary materials can be found at http://www.mdpi.com/1999-4915/10/12/675/s1.
