# Erratum: Johnson et al. Alston Virus, a Novel Paramyxovirus Isolated from Bats Causes Upper Respiratory Tract Infection in Experimentally Challenged Ferrets. *Viruses* 2018, *10*, 675

**DOI:** 10.3390/v13071204

**Published:** 2021-06-23

**Authors:** Rebecca I. Johnson, Mary Tachedjian, Brenton Rowe, Bronwyn A. Clayton, Rachel Layton, Jemma Bergfeld, Lin-Fa Wang, Ina L. Smith, Glenn A. Marsh

**Affiliations:** 1 CSIRO Health and Biosecurity, Australian Animal Health Laboratory, Geelong 3220, Australia; rebecca.johnson@csiro.au (R.I.J.); mary.tachedjian@csiro.au (M.T.); brenton.rowe@csiro.au (B.R.); bronwyn.clayton@ecodev.vic.gov.au (B.A.C.); rachel.layton@csiro.au (R.L.); jemma.bergfeld@csiro.au (J.B.); ina.smith@csiro.au (I.L.S.); 2 Programme in Emerging Infectious Diseases, Duke-NUS Medical School, Singapore 169857, Singapore; linfa.wang@duke-nus.edu.sg

The authors wish to make the following erratum to this paper [1]:

The addition of an author Ina L. Smith due to an omission.

The authors would like to apologize for any inconvenience caused to the readers by these changes.
