# Supplementary material for: Alston Virus, a Novel Paramyxovirus Isolated from Bats Causes Upper Respiratory Tract Infection in Experimentally Challenged Ferrets
Source: Viruses. 2018 Nov 28;10(12):675. doi: 10.3390/v10120675 (PMC6315912; doi:10.3390/v10120675)
Supplement: Supplementary file 1 [file viruses-10-00675-s001.pdf]

**Supplementary material**

Table S1: Comparison of AlsPV and PIV5 untranslated regions and intergenic regions. Differences between AlsPV and PIV5 are in bold and lowercase.

| | | | Length | Boundary Sequence |
|---|---|---|---|---|
| N | 5′ UTR | AlsPV | 102 | AGGCCCGGAA |
| | | PIV5 | **96** | AGG**u**CCGGAA |
| | 3′ UTR | AlsPV | 106 | UUUAAGAAAAAAA |
| | | PIV5 | 106 | UUUAA**ag**AAAAAAA |
| | IGR | AlsPV | 1 | |
| | | PIV5 | 1 | |
| P | 5′UTR | AlsPV | 61 | AGGCCCGGAC |
| | | PIV5 | 61 | AGGCCCGGAC |
| | 3′UTR | AlsPV | 66 | UUUAGAAAAAA |
| | | PIV5 | 66 | UUUAGAAAAAA |
| | IGR | AlsPV | 33 | |
| | | PIV5 | **16** | |
| M | 5′UTR | AlsPV | 33 | AGGCCCGAAC |
| | | PIV5 | **32** | AG**c**CC**ga**A**ca** |
| | 3′UTR | AlsPV | 211 | UUCAAAGAAAA**a** |
| | | PIV5 | **204** | UUCAAAGAAAA |
| | IGR | AlsPV | 22 | |
| | | PIV5 | **23** | |
| F | 5′ UTR | AlsPV | 28 | AGCACGAATC |
| | | PIV5 | 28 | AGCACGAA**c**C |
| | 3′ UTR | AlsPV | 60 | UUUAAGAAAAAA |
| | | PIV5 | **100** | UUUAAGAAAAAA**a** |
| | IGR | AlsPV | 5 | |
| | | PIV5 | **4** | |
| SH | 5′ UTR | AlsPV | 79 | AGGACCGAAC |
| | | PIV5 | 79 | AGGACCGAAC |
| | 3′ UTR | AlsPV | 72 | UUUUAAGAAAAAAA |
| | | PIV5 | **78** | UUUUAA**ag**AAAAAA |
| | IGR | AlsPV | 1 | |
| | | PIV5 | 1 | |
| HN | 5′ UTR | AlsPV | 67 | AGGCCCGAAC |
| | | PIV5 | 67 | AGGCCCGAAC |
| | 3′ UTR | AlsPV | 111 | UUUAAGAAAAA**a** |
| | | PIV5 | 111 | UUUAAGAAAAA |
| | IGR | AlsPV | 13 | |
| | | PIV5 | 13 | |
| L | 5′ UTR | AlsPV | 8 | AGGCCAGA |
| | | PIV5 | 8 | AGGCCAGA |
| | 3′ UTR | AlsPV | 34 | UUUAAGAAAAAA |
| | | PIV5 | 34 | UUUAAGAAAAAA |

Table S2: Comparative growth analysis of AlsPV in multiple mammalian cell lines at 120 h post infection. Significant differences calculated by two-way ANOVA followed by Bonferroni adjustment in GraphPad Prism 5. Non-significant p values represented by 'ns'.

| | **MDBK** | **MDCK** | **PK15a** | **Vero** | **PaKi** | **HeLa** |
|---|---|---|---|---|---|---|
| **MDBK** | | | | | | |
| **MDCK** | ns | | | | | |
| **PK15a** | p<0.01 | ns | | | | |
| **Vero** | p<0.001 | p<0.001 | p<0.05 | | | |
| **PaKi** | p<0.001 | p<0.001 | p<0.05 | ns | | |
| **HeLa** | p<0.001 | p<0.001 | p<0.05 | ns | ns | |

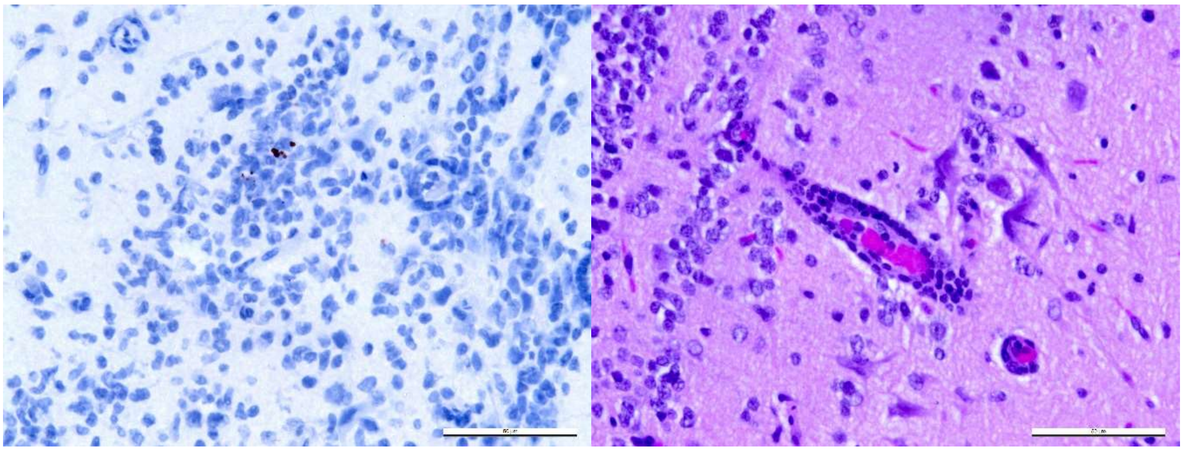

Figure S1: Immunohistochemical and histopathological analysis of olfactory bulb of ferret at 10 days post infection with AlsPV. Olfactory bulb of ferret #10, euthanised on day 10 post infection, was (a) stained with rabbit antiserum against the N protein peptide of AlsPV and (b) assessed by routine H&E staining. Panel (b) shows perivascular cuffing with mononuclear cells, indicating an inflammatory process consistent with viral infection.
